# Supplementary material for: MetaRibo-Seq measures translation in microbiomes
Source: Nat Commun. 2020 Jun 29;11:3268. doi: 10.1038/s41467-020-17081-z (PMC7324362; doi:10.1038/s41467-020-17081-z)
Supplement: Supplementary file 10 — Supplementary Data 7 [file 41467_2020_17081_MOESM10_ESM.zip › File2/Confidence_VeryHigh_Taxonomy/32779_out.krona.html]

Javascript must be enabled to view this page.

members
magnitude
magnitudeUnassigned
count
unassigned
taxon
rank

32779\_out

4

4
superkingdom
2

3
phylum
1239

186801
3
class

order
3
186802

541000
3
family

1263
genus
3

3
species

SRS022071\_contig\_number\_23870SRS053356\_contig\_number\_36047SRS075773\_contig\_number\_11952
1262956

40117
1
phylum

203693
1
class

order
1
189778

189779
1
family

1234
1
genus

species
1
2004993

SRS013951\_contig\_number\_10252
